# Peer-reviewed bioinformatic resources foster accessibility, transparency, reproducibility, and rigor in the microbial sciences

**DOI:** 10.1128/mra.01095-26

**Published:** 2026-10-22

**Authors:** Patrick D. Schloss

**Affiliations:** 1 Department of Microbiology and Immunology, University of Michigan 1259, Ann Arbor, Michigan, USA; Indiana University Bloomington, Bloomington, Indiana, USA

**Keywords:** bioinformatics, special issue, software, databases, web resources

## Abstract

The Databases, Software Tools, and Web-based Services special issue of *Microbiology Resource Announcements* features announcements that foster transparent, open, rigorous, and reproducible practices in the microbial sciences. These resources span the microbial sources and the three types of bioinformatic resources sought in the call for submissions. By publishing in *Microbiology Resource Announcements*, developers improve the discoverability and utility of their bioinformatic resources. Furthermore, it provides them with an opportunity to be credited for their work developing the resource. It also signals to the community that the resource is intended for broader use. The bioinformatic resources announced in this special issue fit alongside the other types of resources more commonly published in the journal and serve as useful companions to research articles published across ASM’s journals portfolio. Looking forward, the journal continues to seek submissions that continue the strong momentum created by the announcements included in this special issue.

## EDITORIAL

Since its creation as the journal *Genome Announcements* in 2013, *Microbiology Resource Announcements* has been a popular venue to publish short manuscripts announcing genome sequences. The journal’s new name signaled to the community a broader scope, including large genomic, metabolomic, and proteomic data sets. This special issue was initiated to call attention to the journal’s desire to publish other microbial resources. Although there had been examples of announcements for databases (e.g., reference 1), software (e.g., reference 2), or data analysis workflows (e.g., reference 3) that enable the analysis of microorganisms, this special issue includes a significant expansion in the number and diversity of resources. It is noteworthy that the tasks performed by the resources included in this special issue represent the growing appreciation that science can be transparent, open, rigorous, and reproducible. After submissions closed for the special issue, we continued to receive inquiries from authors hoping to have an announcement for their bioinformatic resources included in said issue. I sense these inquiries are a real expression of the community’s desire to continue publishing these bioinformatic resources in *Microbiology Resource Announcements*.

A natural question that one might ask is, “Why should I publish an announcement for my bioinformatic resource?” I can think of four reasons. First, it makes a resource discoverable. There are many amazing resources that exist in repositories like GitHub that we use for our research but are not widely known. An announcement article literally announces to the community that such resources exist. Second, citations are frequently used by scientists to measure impact. Having a citable work that is included in the major biographic indexes (e.g., PubMed and Web of Science) helps bioinformaticists get credit in a form that their colleagues and funding agencies understand (4). Third, researchers often use authorship order as a proxy for the level of contribution to a research product (5). By focusing on bioinformatic resources, the authorship order behind a resource may be different from the larger work for which the resource was originally designed. To facilitate this, *Microbiology Resource Announcements* has published manuscripts that are companions to papers published in other ASM Journals (6–8). Finally, many researchers have had the frustrating experience of reading a study only to find that the analysis was performed with “custom R/Python/Perl scripts that are available from the authors.” This has become a shorthand for “please don’t ask to see how the analysis was performed.” In contrast, publishing an announcement signals to the community that this resource is real and meant to be used broadly in the field. At a minimum, the resources published in *Microbiology Resource Announcements* include high-quality documentation that includes demonstrations of using the resource, have software licenses, are attached to a permanent DOI, were created using best practices in software development, and have undergone rigorous peer review. These reasons overlap with justifications for publishing genome sequences, data sets, culture collections, educational materials, or any other microbial resource (6, 9, 10).

Although this special issue is now complete, *Microbiology Resource Announcements* continues to accept bioinformatic resource announcements. If you are looking for exemplars of the quality and rigor of database, software, or web service resources, please consider the announcements included in this special issue.

## EDITOR'S NOTE

**Ed Note**: This article is part of a special series: "Microbial Databases, Software Tools, and Web Services." Patrick D. Schloss serves as Guest Editor for the series, in consultation with the Editor in Chief, Irene Newton. Dr. Schloss's role as the Guest Editor is outside of his duties as the chair of the Journals Committee. To avoid potential conflicts of interest, this article was handled by the Editor in Chief.
